# A new record of *Scedosporium dehoogii* isolated from paddy field soil in Iran: Phylogeny and antifungal susceptibility profiles

**DOI:** 10.32598/CMM.2023.1368

**Published:** 2022-12

**Authors:** Javad Javidnia, Hamid Badali, Iman Haghani, Mahdi Abastabar

**Affiliations:** 1 Department of Medical Mycology, School of Medicine, Mazandaran University of Medical Sciences, Sari, Iran; 2 Invasive Fungi Research Center, Communicable Diseases Institute, Mazandaran University of Medical Sciences, Sari, Iran; 3 Department of Medical Parasitology and Mycology, School of Medicine, Babol University of Medical Sciences, Babol, Iran

**Keywords:** Antifungal susceptibility, Molecular identification, Morphology character-istic, Paddy field soil, *Scedosporium dehoogii*

## Abstract

**Background and Purpose:**

*Scedosporium* species are ubiquitous environmental fungi, which are considered emerging agents that trigger disease in humans and animals. The present study aimed to determine *Scedosporium dehoogii* strain isolated from paddy field soil samples using semi-selective media and evaluate its antifungal susceptibility profile.

**Materials and Methods:**

Three paddy field soil samples were collected during an investigation for the isolation of *Scedosporium* species in Mazandaran province, Iran. Morphological and molecular analyses based on ITS-rDNA sequencing were performed. Furthermore, *in vitro* antifungal susceptibility testing for conventional drugs and novel imidazole (luliconazole) was performed based on Clinical and Laboratory Standards Institute M38-A3 guidelines.

**Results:**

In this study, *S. dehoogii* was isolated from the soil in paddy fields. Based on the results, itraconazole and luliconazole showed the least and most antifungal activity against this isolate, respectively.

**Conclusion:**

Based on the findings, molecular identification was essential for distinguishing the species of *S. dehoogii*. Remarkably, luliconazole showed potent activity against this strain.

## Introduction

*Scedosporium* species are ubiquitous and emerging opportunistic mold pathogens [ 1 ]. They cause invasive fungal infections in otherwise healthy individuals and trigger a broad spectrum of clinical manifestations, including invasive pulmonary disease, mycetoma, osteomyelitis, keratitis, brain abscesses, and meningitis [ 2 , 3 ]. Although *S. apiospermum*, *S. boydii*, *S. dehoogii*, *S. minutisporum*, and *S. aurantiacum* are the main etiological agents of scedosporiosis, some rare species have been reported as etiological agents of this infection [ 1 , 2 ]. 

*Scedosporium* species show reduced susceptibility to all conventional antifungal drugs [ 4 ].

Accurate knowledge of the ecological niches and potential reservoirs of these species has shown that they are more common in human-impacted and polluted environments, such as agricultural soil, public parks, polluted ponds, compost, sewage, polluted water, and effluents of wastewater treatment plants [ 5 - 8 ]. In addition, hydrocarbon-contaminated soil was reported as a habitat of these fungi[ 1 ]. The current study aimed to report the first isolation of *S. dehoogii* from paddy field soil samples and its antifungal susceptibility profiles. Furthermore, the physicochemical parameters of the soil sample were evaluated.

## Materials and Methods

Sampling, fungal isolation, and identification

Three soil samples were collected from 5-10 cm depth of different areas in a paddy field (36.883331, 50.736441) of Ketalem and Sadat Shahr, Ramsar county in Mazandaran province, Iran. The samples were examined in terms of the detection and isolation of *Scedosporium* species. For this purpose, 8 gr soil samples were suspended in 10 ml of sterile distilled water solution, vortexed vigorously and thoroughly, and allowed to settle down. Subsequently, 200 µL of the suspension was inoculated into two semi-selective media, namely Dichloran Rose-Bengal Chloram-phenicol agar supplemented with 10 µg/mL Benomyl [ 9 ] and Scedo-Select III [ 10 ], for up to 2 weeks at 35°C to maximize the selective yield.

## Results

The mimicking *Sceodosporium* species were able to grow in the semi-selective media and were identified by macroscopic and microscopic features followed by molecular identification as previously described [ 9 , 10 ]. Eventually, one *Sceodosporium* species was isolated from two semi-selective media. Morphological features were distinguished on Potato Dextrose Agar (PDA, CONDALAB, Spain) at 35 °C for 7 days. 

**Figure 1:**
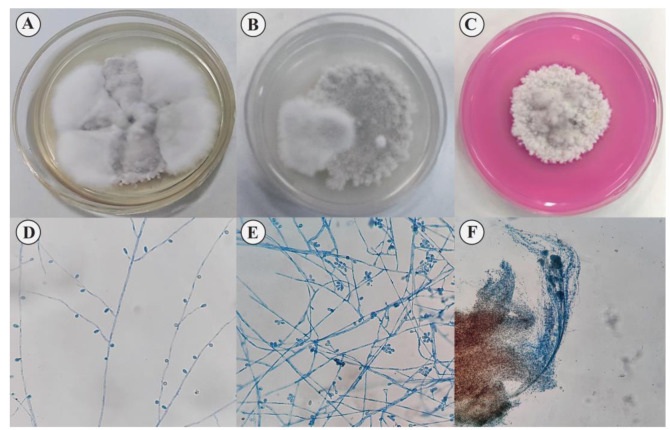
Morphology and microscopic characteristics of *Scedosporium dehoogii*. A) Colony growing on Sabouraud Dextrose Agar B) Potato Dextrose Agar C) Dichloran rose-bengal chloramphenicol agar supplemented with benomyl (10 µg/mL) after 7 days at 35 °C D and E) Conidiogenous cells and cylindrical and sessile conidia F) Synnematous conidiophores were observed to be terminated in a slimy head of conidia.

Colony morphology characteristics of *S. dehoogii* on different media were shown in Figure 1 A-C. The colonies grew and their diameter reached 36 mm after 7 days at 35 °C; they were dense, grayish-white to pale gray, wooly to cottony, whitish irregular border, and yellowish to light-brown reverse (Figure 1B). Microscopic features were studied using lactophenol aniline blue staining (Figure 1 D-F). Solitary conidiogenous cells were usually subhyaline, smooth with thick-wall, generally cylindrical, and ellipsoidal or obovoid conidia (Figure 1 D-E). Synnematous conidiophores were observed to be terminated in a slimy head of conidia (Figure 1 F). The teleomorph form was not observed in these media.

In addition, the final identification of this strain was performed through DNA gene sequencing. Briefly, total genomic DNA was extracted from fresh and pure culture using the phenol-chloroform method [ 11 ]. The polymerase chain reaction was operated using internal transcribed spacer1 (ITS1) and ITS4 primers for the ITS-rDNA regions. Fungal DNA was amplified following a procedure for the initial DNA denaturation stage at 94 °C for 5 min, 35 cycles of DNA denaturation stage at 94 °C for 1 min, annealing stage at 52 °C for 1 min, extension stage at 72 °C for 2 min, and final extension at 72 °C for 5 min [ 9 ]. 

The obtained sequence was adjusted using Lasergene SeqMan software (version 9.0.5) and compared with GenBank (), ISHAM ITS barcoding database (), and Mycobank (). The DNA sequence of the ITS-rDNA region (OP363344) showed 99.82% similarity with the ex-type isolate of *S. dehoogii* (KX664394.1). 

Phylogenetic analysis

**Figure 2:**
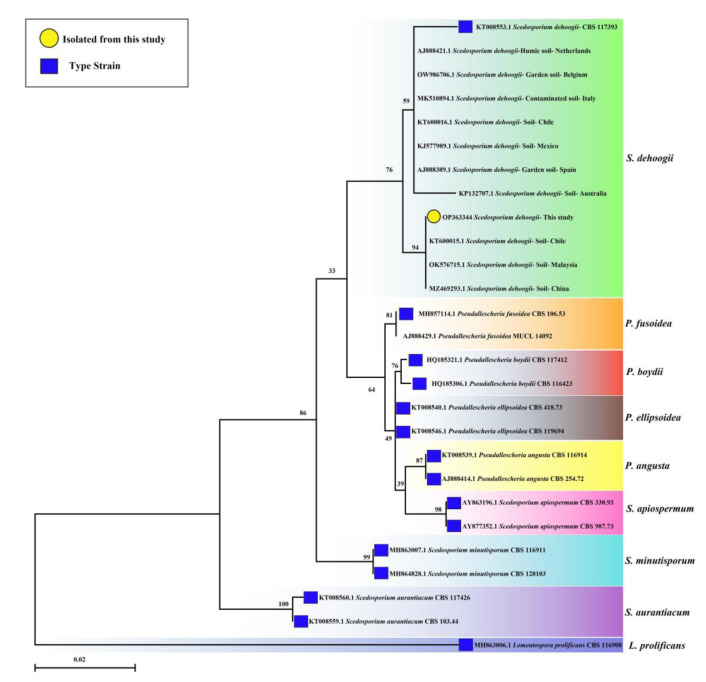
Maximum likelihood phylogenetic analysis of the internal transcribed spacer-rDNA sequence of *Scedosporium dehoogii* obtained from paddy field soil in Iran. Bootstrap values were calculated from 1000 replications. *Lomentospora prolificans* (CBS 116908) was used as the outgroup

Sequences of the ITS-rDNA region of obtained strain and reference strains, including the type strain of *S. dehoogii*, were aligned with the MEGA software (version 11). Maximum likelihood analysis was performed with the Kimura 2-parameter model and 1000 bootstrapping replications (Figure 2). In this phylogenetic tree, 14 different type strains of 8 species in the *Scedosporium* clade were used. Furthermore, 10 strains of *S. dehoogii* from different countries (Belgium, Italy, Chile, Mexico, Spain, Netherlands, Chile, China, Australia, and Malaysia) with soil sources were applied. *Lomentospora prolificans* CBS 116908 was used as the outgroup. 

This Iranian isolate was placed in the *S. dehoogii* clade in this phylogenetic analysis. The sequence of this strain was closely related to strains obtained from Chile (KT600015.1), Malaysia (OK576715.1), and China (MZ469293.1).

Antifungal susceptibility testing

Antifungal susceptibility testing was performed using broth microdilution for filamentous fungi according to the Clinical and Laboratory Standards Institute M38-A3 guideline [ 12 ]. The minimum inhibitory concentrations (MICs) and minimal effective concentrations (MECs) were detected after 72 h and 48 h, respectively. *Paecilomyces variotii* ATCC 22319 and *Candida parapsilosis* ATCC 22019 served as quality controls isolates. 

The MIC/MEC values against amphotericin B, posaconazole, isavuconazole, voriconazole, itraconazole, anidulafungin, micafungin, and luliconazole were 4, 0.5, 4, 4, > 16, 0.032, 0.032, and 0.008 µg/mL, respectively. Among the established antifungal agents, luliconazole was the most active. In contrast, itraconazole was the least active agent against this strain.

Measurements of physicochemical soil parameters

Soil pH, ammonium (NH_4_^+^), and nitrate (NO_3_^-^) concentrations were measured at 7.89, 41.5 mg/kg, and 27.5 mg/kg, respectively, using soil test analysis as described previously [ 13 , 14 ].

## Discussion

Understanding the ecological niches and natural habitats of *Scedosporium* species has usually depended on the sporadic isolation of these emergent fungi. *Scedosporium* species are ubiquitous fungi that can cause pathogenic diseases in humans and animals. In recent reports, several strains of *S. dehoogii* were isolated from sites with high levels of human activity, including playgrounds, public parks, industrial areas, riverbanks, or agricultural areas in different countries, such as France [ 6 ], Australia [ 15 ], Thailand [ 7 , 16 ], Chile [ 9 ], India [ 17 ], Mexico [ 18 ], Sudan [ 19 ], Taiwan [ 8 ], and Austria/The Netherlands [ 5 ]. 

In Iran, several human scedosporiosis have been reported [ 20 - 26 ]. These isolates have been identified based on the morphological and molecular methods that triggered illness through *S. boydii*, *S. apiospermum*, *S. aurantiacum*, and *S. ellipsoideum*, which are the most prevalent species described in the literature [ 20 - 26 ]. The current study reported the presence of *S. dehoogii* in paddy field soil in Iran for the first time. The primary morphological determination was confirmed by molecular methods, which is in agreement with the evidence formerly reported by other studies [ 27 ]. This finding is particularly noteworthy since this species is a potential human and animal pathogen and had not been previously reported in paddy field soil in Iran.

However, there is limited information on the *in vitro* antifungal susceptibility of this fungus. High MECs for echinocandins have been reported in previous investigations [ 28 , 29 ], which is inconsistent with the results of the present study. Based on the findings of the present research, luliconazole was the most active antifungal drug, which is in line with the results of another study [ 30 , 31 ]. However, some studies have reported that *S. dehoogii* is associated with high voriconazole and itraconazole MICs which is in agreement with the findings of this study [ 18 , 29 , 32 ]. 

Soil parameters, like pH and ammonium concentration, have demonstrated predictive usefulness for the isolation of the *Scedosporium* species in soils. Most *Scedosporium* culture-positive soil samples revealed a pH range of 6-8 [ 5 , 18 ]. It has been proven that *Scedosporium* species are strongly linked to environmental organic pollution brought on by animal and human activity [ 5 , 18 , 33 ]. This suggests a typical occurrence of *Scedosporium* species in areas rich in nutrients, fertilized agricultural soils, and human-dominated environments. Knowledge of the ecological niches and natural habitats of *Scedosporium* species is crucial for a better understanding of the dispersion of these fungi and the potential identiﬁcation of an infection source.

## Conclusion

Based on the result, molecular identification was essential for distinguishing the species of *S. dehoogii*. Remarkably, luliconazole has shown potent activity against this strain. However, more comprehensive studies are needed to assess the spectrum of *Scedosporium* species resources and natural niches in Iran.

## Ethical approval

The Ethics Committee approved this research of the Mazandaran University of Medical Sciences (IR.MAZUMS.REC.1398.420).

## Acknowledgments

The authors are grateful to the parasitology and mycology research center staff for their supportive help during the research process. This study was financially supported by a grant (no. 2928) from the School of Medicine, Mazandaran University of Medical Sciences, Sari, Iran.

## Authors’ contribution

H.B. conceived and designed the study. J.Jav., I.H., and J.Jaf. contributed to the acquisition, analysis, and interpretation of data. Material preparation was performed by M.A. and H.B. J.Jav. drafted the manuscript. All authors provided critical revisions for important intellectual content and also read and approved the final manuscript.

## Conflicts of interest

The authors declare no conﬂict of interest.

## Financial disclosure

This study was financially supported by Mazan-daran University of Medical Sciences, Sari, Iran.
